# Contributions to Surgery

**Published:** 1842-02-26

**Authors:** Reynell Coates


MEDICAL EXAMINER.

NEW SERIES.

No. 9.] PHILADELPHIA, FEB. 26, 1842. [Vol. I.

*Contributions to Surgery.* By Reynell Coates, M. D.

Under this head I propose to offer the readers of the Examiner, from time to time, a few notes upon subjects connected with Surgery, many of which could scarcely be treated with advantage in formal papers, though highly important in practice.

### *On the ravages of the fly in wounds and ulcers, and the means of prevention and remedy.*

The occurrence of maggots in wounds and ulcers has been usually attributed to carelessness on the part of the surgeon or assistants; but, without more knowledge of the habits of the fly than surgeons usually possess, it is impossible, in many cases, to avoid the deposition of the eggs, and even with every precaution the accident will occasionally happen. Independent of the disgusting character of the animals, and the alarm occasioned by their presence, the positive irritation produced by them may produce serious complications of cases otherwise simple.

During the prevalence of warm weather, and especially towards the latter part of the summer season, the greatest care is required to prevent the accident; but the danger is by no means confined to the warm months. In the leading original article of the second number of this volume the reader will find it noted that maggots, nearly half an inch in length, infested the wound produced by an operation, late in the month of October, although the patient enjoyed all the advantages of a private lodging with the best attendance, under the directions of some of the neatest and most careful surgeons of the age. I have witnessed similar results in the Pennsylvania Hospital, in wards under my own immediate care, during the severest winter weather; for, in the artificial summer of well warmed apartments, the fly sometimes survives and procreates at all seasons. Many persons suppose that the ordinary dressings, with the turns of the roller usually employed, are sufficient, when faithfully applied, to protect a wound against such inroads; but this is a mistake. The fly rarely, if ever, deposites its eggs upon a bandage soiled with blood when perfectly dry; but when moistened by perspiration, or by the cooling lotions often requisite in cases of local injury, the blood upon the dressings speedily becomes decomposed and fetid by the action of the air. It then furnishes a tempting bed for an insectile *accouchment*. Still more enticing is the purulent discharge with which the bandages are often unavoidably moistened.

The fly generally deposits its eggs, not in the wound, but on the outside of the dressings; and even the odour of suppuration permeating the muslin not unfrequently causes the deposit to be made even upon unsullied bandages. If the bed or the sheets be spotted with the discharges, the eggs may be laid at the distance of some inches from the part; and, as the young worms, when hatched, are capable of recognizing the proximity of their proper food, they immediately make every exertion to reach the injured part. Exceedingly diminutive, at first, the loose texture of a muslin roller opposes no effective barrier to their progress. They penetrate the dressings between the threads, and thus establish themselves in the wound. The daily substitution of fresh bandages is rarely admissible, and therefore it becomes important to devise some sure method of protection against the fly in patients who are compelled to remain at rest. I have never known the maggot to penetrate fine and closely woven linen, which may be employed over the dressings as a guard in some cases; but the young worms may reach the wound by crawling beneath the edges of the linen, unless it be applied very closely to the surface or extended to a considerable distance from the seat of injury.

The generation of maggots is most frequent, for obvious reasons, in injuries of the lower extremities and about the pelvis, or in the sacral ulcerations of the bed-ridden. In compound fractures, or extensive wounds or ulcers of the lower extremities, we have a happy resource in the bran dressing, recommended by Dr. John Rhea Barton, in the treatment of the first named class of accidents. Take the ordinary fracture-box, with its pillow; cover the latter, if you please, with a sheet of thin oiled silk or linen, and having placed the limb—previously dressed if necessary—within the box, fill it completely with bran. This article gives an agreeable support to the member in all directions, and proves an extremely agreeable application to the skin, checking in no inconsiderable degree the tendency to erythema, erysipelas, or excoriation from pressure. It immediately absorbs all discharges and promotes cleanliness. It may be removed from the dressings or the wound at any moment, by simply pushing it aside, and is as readily replaced after the examination. If discharges should have taken place, the soiled portion is found matted firmly into a mass, and may be taken away without changing the remaining clean bran. It often removes the necessity of bandages, and consequent disturbance of the limb; for it will retain a light dressing in place without such aid. In addition to all this, it is, when properly employed, a complete protection against the fly. Eggs may, indeed, be deposited, and maggots bred upon the surface of the bran, when the odour of the discharge is perceptible, but the worms penetrate it very slowly, and to remove them with certainty, it is only necessary to brush away carefully, every day, half an inch of the surface, replacing it with fresh bran.

In compound fractures of the thigh the bran may be employed, when Dr. Physic's apparatus is applied, by simply filling the junct cloth, brought well up towards the groin, employing splints considerably wider than usual. If Dr. Hartshorne's apparatus be preferred —and it is often preferable in compound fractures, because it admits of being constructed with a sliding piece opposite the wound, to facilitate dressing without disturbance*—a narrow sheet should be placed beneath the splints, which may then be filled with bran to any desired extent, and the filling prevented from shifting by placing tow or cotton between the limb and the splints, above and below the parts designed to be protected. These arrangements do not in any degree disturb the permanent extending and counter-extending forces.

In ulceration or mortification of the back from pressure, the bed may be guarded by a piece of oiled silk, and sufficient bran placed upon it to keep off the fly. When the slough is on the sacrum, and the patient lies on a machine-bed, the nurse must be careful to remove the bran from between the nates, and carefully revert the edge of the oiled silk before every stool, if the patient be capable of expressing his wants. It is only in the warmest weather that any precaution against the fly becomes necessary in cases of this class; for the bed-clothing furnishes a sufficient guard at other seasons in parts so difficult of access.

It will happen to the surgeon, occasionally, to be called to cases in which the maggot has possession of the ground already—and the object is to displace it. This is no easy task when the worms are numerous. If but few in number, the only legitimate plan is to remove them, one by one, by means of a forceps. Oil and tar ointment have been strongly recommended as destructive of maggots, by clogging their respiratory spiracles; but, although each of these articles has its proper applications, neither of them is sufficiently powerful to dislodge the worms when numerous and freely moistened with secretions.

When the larvæ of the fly, or any other insects, such as beetles, scolopendra, &c., find their way into the external ear,—an accident by no means uncommon, and horribly painful,—they are easily removed by causing the patient to recline, with the affected ear uppermost, and pouring into the meatus a quantity of sweet oil sufficient to fill the cavity. In a few seconds, or minutes, the intruder, partially suffocated, will present himself on the surface of the fluid, and may be readily removed by the forceps. But, although most insects are destroyed by oil, many of them, and more especially the maggot, will survive its action for a long time. The odour of tar ointment will assist in preventing the attack of the fly on extensive ulcers,—such as those resulting from burns,—but it has very little influence as a remedy for the evil when it has once occurred. In neglected superficial sores of large dimensions, perhaps the best mode of abating the nuisance is to wash the worms away by the force of a jet of water from a large syringe, the bed being protected by floor-cloth or oil-

* I allude here to the original apparatus, without the foot-board and screw since added by that distinguished surgeon,—an alteration which I regard as the reverse of an improvement.

cloth. These vermin are not usually found wandering over the surface, but embed themselves in the granulations, leaving little but their larger extremities visible. By touching their tails somewhat roughly with a spatula or other hard instrument, they will usually detach their probosces, and then yield to the force of the jet much more readily.

About the year 1822, a horrible case of neglect was presented at the Pennsylvania Hospital. The patient was a young negro slave from Maryland or Delaware. He had contracted the venereal disease many weeks before his admission, and a chancre in the groin became infested with the fly. Ignorance, and the dread of punishment, prevented him from seeking proper aid, and the irritation of the maggots caused a most rapid and horrible spreading of the ulceration. On his admission, the whole inside of the left thigh, to within a few inches of the knee, a considerable part of the corresponding surface of the right thigh, the whole scrotum, both groins, the perineum and circumference of the anus, together with a part of the sacral surface, presented one vast, granulating, superficial ulcer, thickly studded in every part with maggots in countless numbers—placed side by side, like thick set pearls, their bodies buried, for nearly their whole length, in the fungous surface, so that when any were removed by force, a cast of their forms remained plainly visibe.

Oil was thoroughly tried in this case without any appreciable result. It scarcely appeared to disturb a single larva. Tar ointment was then employed, but it dislodged but very few of the vermin. Foiled in these attempts, the idea of resorting to a jet of tepid water from a syringe suggested itself, and I succeeded, by this means, in daily washing away an almost incredible number of maggots; so that, in a few days, the ulcer was cleared of them, every where but in the neighbourhood of the anus. Here they had actually succeeded in establishing themselves even within the canal; and those in the immediate vicinity, externally, endeavoured to retreat to the rectum whenever they were disturbed by the water. I found it absolutely impossible to destroy this colony entirely, until I ventured upon repeated injections of spirits of turpentine, regardless of the painfulness of the application. The tar ointment was continued throughout the after treatment, in order to prevent the fresh attacks of the fly; and although the patient was reduced to a state of extreme debility by the extent of surface affected and the gravity of the irritation, the influence of the ointment on the exuberant and flabby granulations was remarkably happy, and, under this treatment, combined with a very gentle mercurial course, the ulcer healed with astonishing rapidity; thus terminating the most shockingly disgusting case which I have ever witnessed, and one in which it was impossible to obtain any assistance, as not a nurse or servant in the establishment was able even to remain in the room during the dressing of the sore.

*Note on the use of Tar Ointment in Burns.*—The effect of the tar ointment upon the granulations, and the smoothness and firmness of the granulations in the foregoing case induced me to test its influence upon wide spread ulcerations from burns. The result was exceedingly happy, and led, as I have been informed by Dr. Thomas T. Hewson, to the extended use of the remedy in the Pennsylvania Hospital, after I ceased to be connected with the institution. The article, as I employed it, was prepared extemporaneously by rubbing together equal parts of fresh tar and basilicon rendered somewhat harder by a slight excess of wax. It appears to be applicable only to burns of a very considerable extent and gravity, and after granulations have formed, when there is not sufficient vital energy in the parts to induce healthy reproduction; as, when the granulations are flat and pale, or soft, exuberant and fungous. It gives them tone, without occasioning the rapid absorption which often follows the application of more powerful stimulants, astringents and escharotics. In burns productive of collapse, the use of the tar should follow the employment of the Kentish ointment, after suppuration is established; and it will often be found to check the profuse and exhausting discharges. I have also seen it produce good effects in extensive erysipelatous inflammations, when the vascular action in the skin is extremely sluggish.

*Note on the influence of the bran dressing in converting compound fractures into simple ones, &c.*—It is now well known that large collections of extravasated blood which have no access to the air, or, in other words, extravasations in internal wounds, are readily absorbed, and rarely give rise to abscess. Even when the quantity of blood is so great as to occasion extensive emphysema in the surrounding cellular tissue from the disengagement of air during coagulation,—an occurrence often witnessed in severe contusions as well as fractures, and attended by crepitation under the pressure of the fingers which may be mistaken for true crepitus by the inexperienced—a consequent abscess is still a rare result. It is very evident that the recoveries without suppuration, occasionally witnessed even in compound luxations of the ankle joint, when the part is speedily covered with a well applied roller without the use of adhesive strips and the bandage becomes agglutinated to the wound and the surrounding skin by a hardened crust of blood, is due to the protection thus given to the coagulum in the wound against the action of the air, which would lead to its decomposition. The wound is placed artificially in the condition of an internal injury; and the process of union between the divided parts is simplified accordingly. The injury resulting from the removal of a scab from the surface of certain ulcers which are prone to the formation of dense superficial crusts is explicable on much the same principle; as is, also, the good effect of the formation of artificial scabs in certain ulcers, by dusting the surface with chalk.

It might be argued that, if the happy results alluded to were due entirely to the exclusion of the atmosphere, the same success would attend the use of oleaginous dressings and adhesive strips, when mad effectually to cover the whole injured surface; which is not found to be the case. Some further remarks will be made on this subject on a future occasion:—at present it is sufficient to mention that these applications usually insure the occurrence of more or less suppuration between the lips of a wound of any considerable extent, because they possess stimulating qualities, either intrinsic or acquired, and thus do not effectually place the part in the condition of an internal wound.

A few years ago I was called, by Dr. Isaac Hays, in consultation on a case of compound fracture of the tibia, with fracture of the fibula also, complicated with two small wounds formed by penetrating fragments of the former bone, which was considerably comminuted. Dr. Hays, in addition to the bandage of strips to the lower part of the limb, employed the bran dressing introduced by Dr. John Rhea Barton, referred to by Dr. Hartshorne in the Hospital Clinical Report contained in this number, allowing the bran to come directly into contact with the wounded surfaces. The oozing blood and the bran were agglutinated into a hard crust over each orifice; as was discovered several days afterwards on removing a portion of the bran for the purpose of examination. The crusts were not disturbed until, after a delay of a number of days, they fell of their own accord, leaving the wounds beautifully cicatrized, and the fracture converted into a simple one, which recovered in the usual time without further difficulty.

I have since recommended the same plan of treatment, with the same definite view, in several cases, and have employed it myself in a few instances of more formidable punctured wounds, with or without fracture, and generally with the same result. Dr. Hartshorne's evidence adds weight to the claims of the practice, and I cannot but regard the general principle as one of great value in vulnerary surgery. Even in more extensive injuries, nothing is lost by the attempt thus to succeed; for, the immediate application of bran is perfectly bland, and, when suppuration supervenes, the article may be washed away with the utmost facility at each dressing, and the equality of pressure and support produced by it, with the facility of removal and examination without disturbance of the part, renders it an invaluable application in the worst forms of lacerated and gun-shot wounds and in compound fractures, in which accidents it has been frequently employed by Dr. Barton.

